# Higher HIV-1 Env gp120-Specific Antibody-Dependent Cellular Cytotoxicity (ADCC) Activity Is Associated with Lower Levels of Defective HIV-1 Provirus

**DOI:** 10.3390/v15102055

**Published:** 2023-10-06

**Authors:** Ryan Yucha, Morgan L. Litchford, Carolyn S. Fish, Zak A. Yaffe, Barbra A. Richardson, Elizabeth Maleche-Obimbo, Grace John-Stewart, Dalton Wamalwa, Julie Overbaugh, Dara A. Lehman

**Affiliations:** 1 Human Biology Division, Fred Hutchinson Cancer Center, Seattle, WA 98109, USA; 2 Department of Microbiology, University of Washington School of Medicine, Seattle, WA 98195, USA; 3 Molecular and Cellular Biology Program, University of Washington, Seattle, WA 98195, USA; 4 Medical Scientist Training Program, University of Washington, Seattle, WA 98195, USA; 5 Department of Global Health, University of Washington, Seattle, WA 98195, USA; 6 Department of Biostatistics, University of Washington, Seattle, WA 98195, USA; 7 Vaccine and Infectious Disease Division, Fred Hutchinson Cancer Center, Seattle, WA 98109, USA; 8 Department of Pediatrics and Child Health, University of Nairobi, Nairobi P.O. Box 30197, Kenya; 9 Department of Medicine, University of Washington, Seattle, WA 98195, USA; 10 Department of Pediatrics, University of Washington, Seattle, WA 98195, USA; 11 Department of Epidemiology, University of Washington, Seattle, WA 98195, USA

**Keywords:** HIV-1, HIV reservoir, antibody-dependent cellular cytotoxicity (ADCC), HIV provirus, pediatric HIV, CS-IPDA, HIV envelope (Env), antibody, defective provirus, intact provirus

## Abstract

A cure for HIV-1 (HIV) remains unrealized due to a reservoir of latently infected cells that persist during antiretroviral therapy (ART), with reservoir size associated with adverse health outcomes and inversely with time to viral rebound upon ART cessation. Once established during ART, the HIV reservoir decays minimally over time; thus, understanding factors that impact the size of the HIV reservoir near its establishment is key to improving the health of people living with HIV and for the development of novel cure strategies. Yet, to date, few correlates of HIV reservoir size have been identified, particularly in pediatric populations. Here, we employed a cross-subtype intact proviral DNA assay (CS-IPDA) to quantify HIV provirus between one- and two-years post-ART initiation in a cohort of Kenyan children (n = 72), which had a median of 99 intact (range: 0–2469), 1340 defective (range: 172–3.84 × 10^4^), and 1729 total (range: 178–5.11 × 10^4^) HIV proviral copies per one million T cells. Additionally, pre-ART plasma was tested for HIV Env-specific antibody-dependent cellular cytotoxicity (ADCC) activity. We found that pre-ART gp120-specific ADCC activity inversely correlated with defective provirus levels (n = 68, r = −0.285, *p* = 0.0214) but not the intact reservoir (n = 68, r = −0.0321, *p*-value = 0.800). Pre-ART gp41-specific ADCC did not significantly correlate with either proviral population (n = 68; intact: r = −0.0512, *p*-value = 0.686; defective: r = −0.109, *p*-value = 0.389). This suggests specific host immune factors prior to ART initiation can impact proviruses that persist during ART.

## 1. Introduction

In its fourth decade of reported cases [1], the ongoing HIV-1 (HIV) pandemic has resulted in roughly 40 million people currently living with HIV, with a disproportionate amount of the global burden located in low- and middle-income countries primarily in sub-Saharan Africa [2]. Though access to antiretroviral therapy (ART) can allow for a near-normal lifespan [3], a cure for HIV remains elusive, as the virus can persist during ART as a latent provirus due to the integration of the HIV genome into that of its host cell. This results in the formation of a reservoir of long-lived, quiescent cells harboring latent HIV provirus capable of host immune evasion and stochastic reactivation, all of which represent a barrier to cure [4,5,6,7]. While the vast majority of persistent provirus during ART is defective [8,9,10] due to large internal deletions or hypermutations [11,12], a fraction of proviruses are intact, of which a subset is able to produce replication competent virus. As a result, cessation of ART ultimately results in rebound of viremia, necessitating life-long administration of ART [5,7,13,14,15].

Despite viral suppression on ART, people living with HIV tend to have increased immune activation and inflammation markers [16,17,18] which are associated with a litany of adverse health outcomes such as cardiovascular disease [19,20], neurological disorders [21], diabetes [22], cancer [23,24], and accelerated aging [25,26,27,28]. Cells harboring HIV provirus, whether intact or defective [10,29,30,31,32,33], are drivers of this chronic immune activation due to their ability to persistently produce and present viral antigens [34], with the size of the reservoir positively associated with inflammation markers [35,36] and risk of comorbidities [21]. Additionally, several studies have demonstrated an inverse relationship between HIV reservoir size and time to viral rebound upon ART interruption, fueling speculation that a functional cure for HIV may be possible by limiting the reservoir’s size [37,38,39,40]. Though the size of the reservoir that persists on ART can vary significantly between people, its rate of decay is low across individuals [41,42]. Thus, it is important to identify correlates of a reduced HIV reservoir and to develop interventions to mediate such a reduction [43].

The notion that an exceedingly low reservoir size can result in a functional cure has received considerable attention in the setting of pediatric HIV infection. There have been several reported cases of children with extremely small reservoirs who initiated ART during very early life who maintained viral suppression for years after ART interruption, perhaps the most notable being the “Mississippi baby” [44,45,46,47]. Larger studies corroborate these findings, revealing that ART initiation within the first year of life is beneficial for limiting pediatric reservoir size [46,48,49]. While early ART initiation in children is common in high resource settings like Europe and North America, children in sub-Saharan Africa, who represent the majority of children living with HIV, typically begin ART during chronic HIV infection [50]. Initiation of ART during chronic infection in children results in a latent reservoir size comparable to that observed in adult populations [51], with long-term ART in children also associated with adverse health outcomes and increased immune activation [52,53,54]. Yet studies of the pediatric HIV reservoir, especially those related to its establishment during chronic HIV infection, remain limited compared to those in adults. Additionally, despite representing a smaller fraction of global HIV cases, most studies on this subject are conducted in resource-rich countries where HIV subtype B is dominant [55,56]. Thus, further work to understand factors that influence the HIV reservoir in pediatric cohorts, particularly in cohorts representing populations where HIV prevalence is highest and the dominant circulating strains are non-subtype B, is crucial [57,58,59].

Although HIV integrates its genome into host cells during viral replication throughout untreated infection, the majority of archived proviral sequences that persist during ART appear to be seeded near the time of ART initiation, as they are typically genetically similar to those of circulating viruses at that time [60,61,62]. This suggests that factors present at ART initiation may impact reservoir seeding and composition and that interventions targeting the HIV reservoir may be advantageous to initiate at this critical time. The host immune response has been implicated as one such factor. For example, studies have shown that cells harboring latent provirus exhibit resistance to CD8+ T cell killing suggesting selection for CTL escape [63,64,65]; however, CD8+ T cell depletion in macaques at ART initiation does not impact the size of the established SIV reservoir [66]. Conversely, broadly neutralizing antibodies (bNAbs) have been shown to prolong viral suppression after ART interruption in humanized mice [67,68], nonhuman primates [69,70], and humans [71,72,73,74,75]. This suggests that in addition to neutralizing capabilities, these administered antibodies may also reduce the latent reservoir. Indeed, animal models have demonstrated the ability of bNAbs to clear HIV-infected cells [73] and even interfere with reservoir establishment through Fc-FcR-mediated mechanisms [67], implying that Fc-mediated effector functions, including antibody-dependent cellular cytotoxicity (ADCC), could influence reservoir establishment.

While these studies focus on the effect of heterologous antibody therapies, fewer studies have investigated the ability of antibodies induced by natural infection to impact the HIV reservoir. A recent study showed that contemporaneous antibodies blocked the viral outgrowth of a majority of viruses in the latent reservoir [76], indicating the ability of infection-induced antibodies to recognize and inhibit reactivated virus. Additionally, ex vivo studies have demonstrated the ability of autologous antibodies to mediate ADCC against paired reactivated CD4+ T cells [77,78,79]. Together, these studies suggest autologous antibodies may influence the HIV reservoir through mechanisms such as ADCC. HIV Env-specific ADCC activity has previously been shown to correlate with clinical outcomes including HIV transmission/acquisition [80,81,82,83], survival [83,84,85,86,87], and elite controller status [88,89,90,91]. However, no studies to date have evaluated the association between HIV infection-induced ADCC and proviral DNA levels that persist during ART.

In this study, we tested the hypothesis that HIV Env-specific ADCC activity at the time of ART initiation inversely correlates with the size of the established HIV reservoir. We leveraged samples from the Pediatric Adherence Diary (PAD) study [92] that enrolled ART-naïve children in Kenya living with HIV with longitudinal samples collected over several years during continuous ART. Using the rapid and fluorometric ADCC (RFADCC) assay [93] that was previously shown to correlate with pediatric clinical outcomes [84,85], and the newly developed cross-subtype intact proviral DNA assay (CS-IPDA) [94], this study investigated the relationship between pre-ART ADCC activity and levels of HIV provirus in children during ART. Our findings suggest that higher HIV Env gp120-specific ADCC activity in plasma at the time of ART initiation may reduce total and defective provirus levels during ART, but not the intact reservoir.

## 2. Materials and Methods

### 2.1. Cohort

Between 2004 and 2005, the Pediatric Adherence Diary (PAD) study enrolled ART-naïve children living with HIV in Nairobi, Kenya, aged 18 months to 12 years, in a longitudinal clinical trial to evaluate adherence diaries during ART [92]. The study provided ART at the enrollment visit to 103 children, and blood samples were collected at enrollment and again every three to six months during the first two years after ART initiation for those who remained in follow-up. The study was approved by the University of Washington and Fred Hutchinson Cancer Center Institutional Review Boards and Kenyatta National Hospital Ethics and Research Committee. Caregivers provided written informed consent for their children’s participation and for the use of banked samples in future studies.

### 2.2. HIV RNA/DNA Measurement

HIV RNA was previously measured in longitudinal plasma samples using Gen-Probe HIV RNA assay with a lower limit of detection of 150 copies/mL [95]. PBMC samples were selected for HIV DNA quantification from timepoints between 12- and 24-months post-ART initiation that had viral suppression (HIV RNA levels < 1000 copies/mL). From the original cohort (n = 103), samples that met this inclusion criteria were available for 72 participants, with up to two PBMC samples available per child. If two PBMC samples from the same child met the inclusion criteria, we quantified HIV provirus in both samples and averaged the two measures. To account for the diverse subtypes of HIV circulating in Kenya [96], CS-IPDA [94] was used to measure the number of total and intact proviral copies isolated from cryopreserved PBMCs. Low-shearing genomic DNA extraction and CS-IPDA were performed as previously described [97]. CS-IPDA reactions were completed in triplicate with additional replicates performed on samples with no detectable intact provirus until either intact provirus was detected or a minimum of 10^5^ cells were interrogated. The CS-IPDA can detect a single copy of intact provirus; samples with undetectable intact provirus were set to 0.5 copies over the number of cells interrogated normalized to 10^6^ cells. In a prior analysis, less than 1% of intact sequences were incorrectly classified as defective, which suggests that underestimating intact provirus because of sequence diversity is rare [94]. The number of defective proviral copies was determined by subtracting the number of intact proviral copies from the total number of proviral copies. Intact provirus levels were only measured if samples had ≤40% DNA shearing as measured by the RPP30 reference assay [97,98]. Thus, total provirus data were included in our analysis for all 72 participants, while intact and defective HIV proviral copies were included for 65 participants that had DNA shearing rates ≤40%.

### 2.3. Rapid and Fluorometric ADCC Assay

The rapid and fluorometric ADCC (RFADCC) assay, which has been associated with clinical outcomes [82,84,85] and is correlated with results from different ADCC assays [85], was performed as previously described [85,93] to measure HIV Env-specific ADCC activity at study enrollment (time of ART initiation) using plasma samples heat-inactivated at 56 °C for 1 h. Briefly, the cytosols of CEM.NKR cells (NIH AIDS Reagent Program, Catalog #458) were stained with CFSE dye (Vybrant CFDA-SE. Cell Tracer Kit, Invitrogen, Waltham, MA, USA) followed by cellular membrane staining with either CellVue Claret Far Red cell linker dye (Sigma Aldrich, Saint Louis, MO, USA) or PKH26 cell linker dye (Sigma Aldrich). These double stained cells were then coated with either Clade A BG505.W6M.ENV.B1 gp120 (Cambridge Biologics, Brookline, MA, USA; GenBank: ABA61515), Clade A/D BL035.W6M.ENV.C1 gp120 (Immune Tech, New York, NY, USA; GenBank: DQ208480), or Clade C ZA.1197MB gp41 (Immune Tech; GenBank: AY463234) antigen at a ratio of 1.5 ug of antigen per 100,000 cells for one hour at room temperature. The clade A BG505 gp120 antigen, which was derived from a Kenyan infant living with untreated HIV infection, is the dominant HIV clade circulating in Kenya [96], and the clade A/D recombinant BL035 gp120 antigen has previously been shown to be representative of gp120 from diverse clades when used in the RFADCC assay [84]. The ZA.1197MB gp41 antigen represents one of the few gp41 antigens derived from a primary isolate. During the one-hour coating step, plasma samples were diluted to either 1:100,000 if BG505 gp120 was the coating antigen or 1:32,000 if BL035 gp120 or ZA.1197 gp41 was the coating antigen. These dilutions were experimentally determined to provide the best separation of measurements across samples from this specific cohort for each individual antigen while also avoiding a prozone effect. Additionally, monoclonal antibodies serving as positive controls were diluted to 100–500 ng/mL, and an Anti-HIV Immune Globulin (HIVIG, NIH ARP, Catalog #3957) positive control and a Human Negative Control Serum (NIH ARP, Catalog #2411) were both diluted to a 1:5000 dilution. All samples were diluted in RPMI containing penicillin (100 U/mL), streptomycin (100 µg/mL), amphotericin B (250 ng/mL), L-glutamine (2 mM), and fetal bovine serum (10%) (RPMI complete). Following the one-hour antigen coating step, the double stained cells were washed, and a total of 5000 double-stained, coated target cells were added to 100 µL of each plasma or control dilution in duplicate in a 96 well U bottom TC-treated plate (Corning, Corning, NY, USA). The target cells and plasma dilutions were mixed and then incubated for 15 min at room temperature, followed by the addition of 250,000 PBMCs from a seronegative donor for an effector to target cell ratio of 50:1. These cells were then left at 37 °C for four hours to allow for RFADCC activity to occur and then washed and fixed in 2% paraformaldehyde (Santa Cruz Biotechnology, Dallas, TX, USA). The next day, RFADCC activity was measured via flow cytometry (BD Symphony). The CFSE, CellVue, and PKH26 dyes were detected in the FITC, APC, and PE channels, respectively. The collected data were then analyzed using FlowJo (v.9.9, Treestar). ADCC was determined as the percentage of either PKH or CellVue-positive, CFSE-negative cells out of the total PKH, or CellVue-positive cells after subtracting for background activity. Background ADCC was determined as the ADCC activity of media against uncoated target cells, which was set to 3–5%. All data were then normalized to the average ADCC activity measured in the HIVIG positive control wells. Three biological replicates, each consisting of two technical replicates, were performed for each antigen. For BG505 gp120 and ZA.1197 gp41 antigens, all three biological replicates were performed using seronegative PBMC donor cells from a different donor to assure that the results were not specific to a particular PBMC donor. The three biological replicates carried out for the BL035 gp120 antigen were performed using PBMCs from two different seronegative donors. The results for both BG505 and BL035 gp120 antigens across all replicates were averaged for each child to report one gp120-specific percent ADCC.

### 2.4. Statistical Methods

To reduce skewness, all HIV provirus and ADCC data were log transformed for analyses. Pearson correlation coefficients were generated to test for associations between each proviral category and both gp120 and gp41-specific ADCC activity. ADCC data were also stratified into two groups labeled either “ADCC ≥ Median” or “ADCC < Median” based on a participant’s plasma ADCC activity in relation to the cohort median. The ADCC data were additionally stratified into two groups labeled “ADCC High” for those in the highest quartile of ADCC activity or “ADCC Low” for those in the lowest quartile. Student’s T-tests were performed to test for differences in the mean copy number of HIV provirus between ADCC groups. Multivariable linear regression models with backward stepwise selection were used to assess potential predictors of each category of HIV provirus and address confounding effects. Age at ART start, a proxy for time to ART, was included in the model given its established clinical significance. Other potential predictors included pre-ART CD4 percent, pre-ART viral load (log_10_ copies/mL), gp120 ADCC levels, and gp41 ADCC levels. A pre-determined cutoff for statistical significance was set at a *p*-value of ≤0.05, with 0.05 < *p*-value ≤ 0.01 deemed a trend. All analyses were performed using GraphPad Prism Version 9.5.0 or R version 4.0.4 (R Core Team 2021).

### 2.5. Cell Lines

In the RFADCC assay, CEM.NKR cells (RRID: CVCL_X622; originally derived from female human T-lymphoblastoid cells) were used as target cells. These cells were obtained from the NIH AIDS Reagent Program (cat #: 458) and maintained at 37 °C in RPMI 1640 media with penicillin (100 U/mL), streptomycin (100 µg/mL), amphotericin B (250 ng/mL), L-glutamine (2 mM), and fetal bovine serum (10%) added. We did not further authenticate the cells.

## 3. Results

### 3.1. Study Population and Baseline Characteristics

Children living with HIV in Nairobi, Kenya were provided ART at enrollment into the Pediatric Adherence Diary (PAD) study [92] and monitored through two years of longitudinal follow-up visits. For this study, plasma and PBMC samples that met our inclusion criteria, described in Materials and Methods, were available from 72 of the participating children. The median age at study enrollment was 4.92 years, ranging from 1.29 to 12.7 years. Pre-ART median viral load in this cohort was 5.96 log_10_ copies/mL (min: 4.18, max: 6.96), with median CD4 percent at 6.30% (min: 0.700%, max: 73.4%) and a median CD4 count of 354 cells/mm^3^ (min: 15.0, max: 2009). Both CD4% and CD4 count are reported because in newborns, the absolute number of T cells is much higher than in adults and gradually decreases to adult-like levels between the ages of six and 12; thus, CD4% is used when comparing children of varying ages [99,100]. Of the children in our study with CD4% data at study enrollment, 79% (n = 45) had a CD4% < 15%, which is considered immunosuppressed, and 21% (n = 12) were not immunosuppressed (CD4 ≥ 15%). Participants assigned female at birth represented 54% (n = 39) of the cohort, with those assigned male representing 46% (n = 33). Following study enrollment, nearly all participants (n = 70) started an ART regimen consisting of one non-nucleoside reverse transcriptase inhibitor (NNRTI) and two different nucleoside reverse transcriptase inhibitors (NRTI), with one participant receiving a triple NRTI regimen and one receiving a combination of one NNRTI, one NRTI, and one protease inhibitor (Table 1).

### 3.2. Quantifying Persistent HIV Provirus

**Figure 1:**
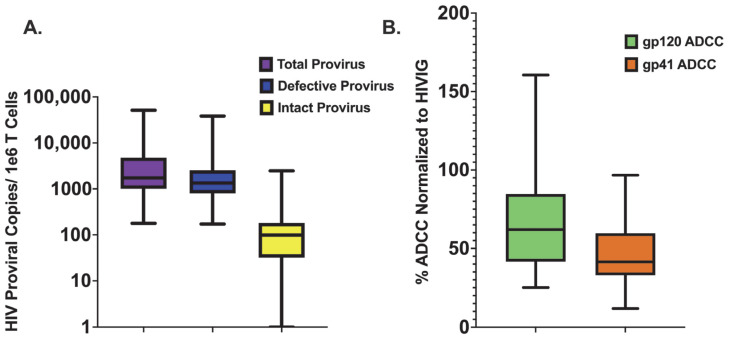
**Distribution of HIV Proviral Copies and ADCC Activity Measured Across the Cohort.** (**A**). Distribution of the number of HIV proviral copies measured across the study cohort for total (**left**) (n = 72), defective (middle) (n = 68), and intact (**right**) (n = 68) provirus; (**B**). Distribution of ADCC activity measured across the study cohort for both gp120 (**left**) (n = 72) and gp41 (**right**) (n = 72).

The CS-IPDA [94], a three-target digital droplet PCR, was used to measure the number of intact and total proviruses per one million CD4+ T cells using available cryopreserved PBMCs obtained between 12 and 24 months post-ART initiation during viral suppression. The number of defective proviruses, defined as those lacking at least one region of the genome detected by CS-IPDA, was determined by subtracting the number of intact from the total HIV proviral copies. Though this assay cannot directly measure replication competence and may overestimate reservoir size if defects in the genome occur outside of the probed regions, intact (containing all three targets) provirus is used as a proxy measure for the replication competent reservoir, as IPDA measurements correlate with results from the quantitative viral outgrowth assay (QVOA) [101]. Due to the QVOA’s requirement for a high volume of sample input, the CS-IPDA was employed, as it is a high-throughput assay that requires less sample volume, can interrogate provirus across clades, and has an absolute limit of detection of one copy per reaction [94]. In this study, there was a considerable range in the number of proviral copies per one million CD4+ T cells measured for total (median: 1729, min: 178, max: 5.1 × 10^4^), intact (median: 99, min: 0, max: 2469), and defective (median: 1340, min: 172, max: 3.84 × 10^4^) provirus (Figure 1A). As has previously been observed [8,9,10], the vast majority of detected provirus was defective (mean % defective: 86.3%, range: 6.5–100%).

### 3.3. HIV Env gp120-Specific ADCC Activity Is Inversely Associated with Levels of Defective Provirus

Most of the HIV reservoir is established near the time of ART initiation [60,61,62]; thus, plasma ADCC activity against HIV Env gp120 was evaluated at study enrollment just prior to ART start. We independently tested two gp120 antigens, BG505 and BL035, which were both derived from Kenyan infants living with HIV. The average ADCC activity for each participant measured against BG505 was highly significantly correlated with ADCC activity measured against BL035 (r = 0.773, *p*-value = <0.0001) (Supplementary Figure S1A). The results for both antigens across three replicates were averaged for each child to report one gp120-specific percent ADCC. Median cohort ADCC activity against gp120 was 62.0%, ranging from 25.1% to 161% when normalized to the activity of a standard HIV plasma pool (Figure 1B).

**Figure 2:**
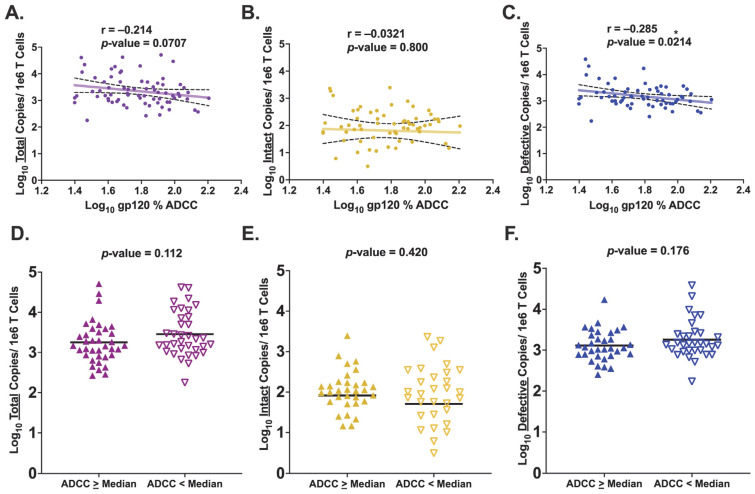
**Association Between gp120-Specific ADCC Activity and Levels of HIV Provirus.** (**A**–**C**) Pearson correlation of log_10_ gp120-specific ADCC activity and copies of HIV provirus measured for each proviral category. The best fit line and its 95% confidence interval are imposed onto each graph. (**A**) Total HIV provirus (n = 72); (**B**) Intact HIV provirus (n = 68); (**C**) Defective HIV provirus (n = 68); (**D**–**F**) Unpaired *t* test comparing mean log_10_ HIV proviral copies between “ADCC ≥ Median” and “ADCC < Median” groups. Participants were stratified into either group based on a participant’s ADCC activity in relation to the cohort median; Participants at, or above, cohort median were labeled “ADCC ≥ Median” with those below the cohort median labeled as “ADCC < Median.” (**D**) Total HIV provirus (n = 72); **(E)** Intact HIV provirus (n = 68); (**F**) Defective HIV provirus (n = 68). Different colors in the figures designate which proviral category is being analyzed: purple: total; yellow: intact; blue: defective. In figures D–F, the ADCC ≥ Median group is denoted by filled in triangles pointing upward, with the ADCC < Median group denoted by empty triangles pointing downward. * denotes a *p*-value ≤ 0.05.

To test our hypothesis that pre-ART HIV Env-specific ADCC activity is inversely associated with the size of the HIV reservoir, we first examined how pre-ART gp120-specific ADCC activity correlated with levels of total HIV provirus during ART. The results demonstrated a trend for an inverse association between HIV Env gp120-specific ADCC activity and the level of total provirus that persists during ART (r = −0.214, *p*-value = 0.0707) (Figure 2A). Since total HIV provirus comprises both intact and defective proviruses, we repeated the analysis for these two proviral categories separately. Based on studies demonstrating faster natural decay of cells harboring intact compared to defective provirus on long-term ART [102,103,104,105], we anticipated an inverse association with gp120-specific ADCC activity and the size of the intact reservoir but not with levels of defective provirus. To our surprise, there was no observed association between the size of the intact reservoir and gp120-specific ADCC activity (r = −0.0321, *p*-value = 0.800) (Figure 2B). In contrast, the results did demonstrate a statistically significant, moderate inverse association between gp120-specific ADCC activity and levels of defective HIV provirus (r = −0.285, *p*-value = 0.0214) (Figure 2C). We also performed these same analyses with a more restrictive inclusion criteria to only include reservoir measurements from samples taken with viral suppression both at reservoir measure as well as for six months prior. This yielded similar results, demonstrating a trend for an inverse association between gp120-specific ADCC and levels of defective provirus (Pearson correlations: total: r = −0.155, *p*-value = 0.208; intact: r = 0.0194, *p*-value = 0.881; defective: r = −0.219, *p*-value = 0.0867). In this analysis, a decrease in statistical significance was observed, potentially due to a decrease in statistical power (total: n = 68 versus n = 72; intact and defective: n = 62 versus n = 65).

To further examine these associations, each participant was stratified based on ADCC activity relative to the median ADCC activity of the cohort. The two groups were labeled either “ADCC ≥ Median” or “ADCC < Median.” T-tests were performed to assess differences in mean HIV proviral copies between the two groups. This analysis did not demonstrate significantly fewer total HIV proviral copies, on average, in the ADCC ≥ Median compared to the ADCC < Median group (*p*-value = 0.112) (Figure 2D). There was also no significant difference for the mean number of intact proviral copies between the two groups (*p*-value = 0.420) (Figure 2E). Additionally, the ADCC ≥ Median group also did not demonstrate statistically significantly fewer defective proviral copies compared to the ADCC < Median group, though the results appeared to potentially be in that direction (*p*-value = 0.176) (Figure 2F). To further separate those with high and low ADCC, a binning approach was performed by including participants with ADCC activity at, or above, the cohort 75th percentile into an “ADCC High” group (n = 18) and participants with ADCC activity at, or below, the 25th percentile into an “ADCC Low” group (n = 18). The results demonstrated no change in statistical significance for total provirus (*p*-value = 0.254) or intact provirus (*p*-value = 0.280) but did demonstrate a shift to a statistically significant difference between the two groups for mean defective proviral copies (*p*-value = 0.0232) despite the smaller sample size (Supplemental Figure S2A–C).

To address potential confounding effects, we ran univariate analyses to test for an association of age at ART start, pre-ART viral load, CD4%, and CD4 count, independently, with the levels of each proviral category. While we do not know the exact timing of seroconversion for each child, we assume vertical transmission near the time of birth and use participant age at ART start as a proxy for time to ART initiation. In univariate analysis, none of the potentially confounding factors we analyzed were significantly associated with the levels of any proviral category in our cohort. However, as several studies have reported an association with time to ART initiation and the size of the HIV reservoir in children [45,106,107], we performed a multivariate linear regression controlling for time to ART initiation in the model. This analysis also demonstrated a moderate inverse correlation of gp120-specific ADCC activity with levels of defective HIV provirus in both a univariate linear regression (coefficient = −0.635, *p* = 0.0202) and a multivariate model controlling for age at ART start (coefficient = −0.588, *p* = 0.0414) (Supplementary Table S1). Taken together, the results suggest increased ADCC activity against HIV Env gp120 may be associated with a reduced number of defective, but not intact, copies of HIV provirus during ART.

### 3.4. HIV Env gp41-Specific ADCC Activity Is Not Associated with Levels of Persistent HIV Provirus

**Figure 3:**
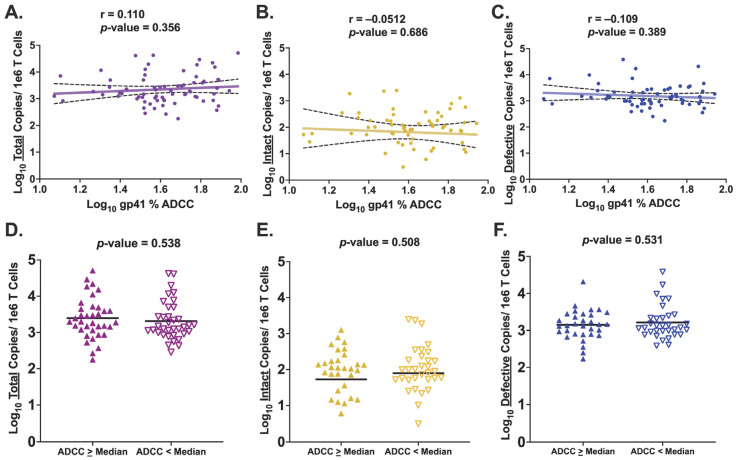
**Association Between gp41-Specific ADCC Activity and Levels of HIV Provirus.** (**A**–**C**) Pearson correlation of log_10_ gp41-specific ADCC activity and copies of HIV provirus measured for each proviral category. The best fit line and its 95% confidence interval are imposed onto each graph. (**A**) Total HIV provirus (n = 72); (**B**) Intact HIV provirus (n = 68); (**C**) Defective HIV provirus (n = 68); (**D**–**F**) Unpaired *t* test comparing mean log_10_ HIV proviral copies between “ADCC ≥ Median” and “ADCC < Median” groups. Participants were stratified into either group based on a participant’s ADCC activity in relation to the cohort median; Participants at, or above, cohort median were labeled “ADCC ≥ Median” with those below the cohort median labeled as “ADCC < Median.” (**D**) Total HIV provirus (n = 72); (**E**) Intact HIV provirus (n = 68); (**F**) Defective HIV provirus (n = 68). Different colors in the figures designate which proviral category is being analyzed: purple: total; yellow: intact; blue: defective. In figures D–F, the ADCC ≥ Median group is denoted by filled in triangles pointing upward, with the ADCC < Median group denoted by empty triangles pointing downward.

Since HIV Env consists of both gp120 and gp41, we next wanted to determine if gp41-specific ADCC activity demonstrated the same associations with levels of HIV provirus as those observed for gp120-specific ADCC. Median gp41-specific ADCC activity across the cohort was 41.4%, ranging from 11.8% to 96.7% (Figure 1B). The ADCC activity specific for gp41 strongly correlated with the ADCC activity specific for gp120 (r = 0.511, *p*-value < 0.0001) (Supplemental Figure S1B). However, there was no statistically significant association observed between gp41-specific ADCC activity and total proviral levels (r = 0.110, *p*-value = 0.356) (Figure 3A). Examining each individual proviral category, we again observed no significant association between gp41-specific ADCC activity and neither the size of the intact reservoir (r = −0.0512, *p*-value = 0.686) (Figure 3B) nor the number of defective proviral copies (r = −0.109, *p*-value = 0.389) (Figure 3C). Multivariate linear regression analyses controlling for potential confounding effects, as described above, for gp41-specific ADCC activity reported similar results (Supplementary Table S1). When the analyses were run with the inclusion criteria requiring viral suppression at the time of HIV DNA quantification as well as for six months prior, again we did not observe a significant change in the reported results (Pearson correlation: total: r = 0.150, *p*-value = 0.223; intact: r = −0.0185, *p*-value = 0.887; defective: r = −0.0720, *p*-value = 0.578).

Employing the previously described binning approach in relation to the cohort median gp41-specific ADCC activity, no significant difference was observed between the two groups for mean total (*p*-value = 0.538), intact (*p*-value = 0.508), or defective (*p*-value = 0.531) copies of HIV provirus (Figure 3D–F). When binning was performed based on a participant’s relation to the cohort 75th and 25th percentile, there was no change in the observed statistical significance for any of the proviral categories (total: *p*-value = 0.242; intact: *p*-value = 0.742; defective: *p*-value = 0.770) (Supplementary Figure S2D–F). Therefore, in contrast to what was observed with gp120-specific ADCC activity, ADCC activity against HIV Env gp41 at the time of ART initiation is not associated with levels of any proviral category. These contrasting findings suggest a unique role for ADCC-mediating antibodies specific for gp120 to impact the persistent proviral landscape during ART.

## 4. Discussion

The cytolytic nature of host immune-mediated effector functions such as ADCC supports the potential for antibody responses present prior to ART to impact the establishment of the HIV reservoir, yet our understanding of this remains limited. To address this, we assessed the association between pre-ART HIV Env-specific ADCC activity and levels of persistent HIV provirus. The results suggest a moderate inverse correlation between gp120-specific, but not gp41-specific, ADCC activity and levels of defective persistent provirus. The moderate nature of this observed association may reflect the fact that several factors presumably impact establishment of persistent HIV provirus, with our results implicating gp120-specific ADCC as one of these factors. Interestingly, we did not observe this same association with the size of the intact reservoir, which adds to the mounting evidence of potential differences in kinetics between these two proviral populations [101,102,104,105].

HIV Env is presented on the surface of reactivated latent cells largely as gp120 or gp41 monomers [108] and is the main HIV protein targeted by ADCC-mediating antibodies [109,110], with ADCC epitopes predominately exposed when Env binds CD4 within the same infected cell [78,79,110,111]. Importantly, HIV uses its Nef and Vpu proteins to downmodulate cell surface levels of CD4, decreasing Env-CD4 interactions and limiting exposure of ADCC epitopes [110,112,113]. Thus, one hypothesis to explain the differences we observed in associations between gp120-specific ADCC and intact, versus defective, HIV provirus is that a defective provirus with nonfunctional Nef and/or Vpu could result in suboptimal CD4 downregulation, promoting exposure of Env-CD4-induced epitopes on a cell’s surface and increasing susceptibility to ADCC-mediated clearance [114,115,116]. Studies probing the detailed structure of defective proviruses and the corresponding functionality of their Nef and Vpu genes would be needed to test this hypothesis.

While epitope targets capable of mediating ADCC are found in both gp120 and gp41 subunits [117,118], gp41-specific ADCC activity did not significantly correlate with levels of persistent provirus in our study. The Env trimer regularly sheds its gp120 subunit, leaving a gp41 stump displaying an immunodominant epitope on its ectodomain [119,120], which is the primary antibody target during acute HIV infection [120,121]. However, due to its highly variable nature, gp120 continually escapes antibody responses during chronic HIV infection, promoting a broad polyclonal antibody response resulting in gp120 becoming the dominant antibody target [120,122,123,124]. This could similarly lead to increased ADCC activity targeting gp120 versus gp41. Thus, one hypothesis to explain the differences we observed in associations with HIV provirus levels and gp120-specific ADCC compared to gp41-specific ADCC activity is that there is a greater quantity, and quality, of antibodies targeting gp120 compared to gp41.

Our results demonstrating an inverse association between gp120-specific ADCC activity and levels of defective HIV provirus are particularly interesting given that ADCC-mediating antibodies specific for the V2 region in gp120 were identified as correlates of protection in the moderately successful RV144 vaccine trial [81]. This finding is notable as therapeutic vaccines intended to mediate clearance of cells harboring HIV provirus are currently beginning to enter clinical trials [125,126]. The results of this study provide evidence to support consideration of ADCC activity as an immune outcome measure in these trials. While our observation that gp120-specific ADCC activity inversely correlates only with levels of defective provirus, an intervention capable of targeting cells with defective provirus could still prove useful in decreasing the overall number of cells capable of contributing to chronic immune activation during long-term ART [10,29,30,31,32,33].

There are several limitations to our study, the most relevant being that the investigated cohort included a wide age range at time of ART initiation. This is pertinent because for children living with untreated HIV infection, by the age of two, there is approximately a 50% mortality rate [58,59,127], reaching 80% by the age of five [128]. Thus, the children in this study include the roughly 20% of children who lived past age five despite untreated HIV infection. Therefore, the cohort studied here may represent a unique population with less applicability to the broader population. This study also had several unique strengths, including access to pediatric samples beginning at the point of ART initiation. By leveraging the CS-IPDA, this study investigated intact and defective proviruses separately, which is important as recent studies have demonstrated differences in population dynamics between these two proviral populations such as a significantly higher decay rate of intact proviruses compared to defective proviruses during long-term ART [101,102,104,105]. Additionally, the CS-IPDA allowed for the study of a cohort from Kenya, where HIV subtypes A and D are most prevalent, whereas subtype B has been the primary focus of most reservoir studies up to this point despite representing a relatively small fraction of the global HIV burden [56].

To date, the most relevant factor associated with the size of the established reservoir is time between primary HIV infection and ART initiation. Here, we observe gp120-specific ADCC activity inversely associates with the levels of defective HIV provirus during ART with a similar magnitude of association to that of time to ART initiation [129]. These findings suggest that host immune effector functions may limit the number of cells harboring defective HIV provirus during ART. Additionally, these data support the idea that the dynamics of cells harboring intact HIV provirus differ from those harboring defective HIV provirus and thus should be studied, and treated, individually.

In summary, our data suggest that HIV gp120-specific antibodies capable of mediating ADCC may reduce the established levels of defective, but not intact, persistent HIV provirus. Importantly, this represents a factor that can be manipulated via biomedical interventions and thus could be of interest as a possible strategy to augment ART. Therefore, further studies on the impact of ADCC-mediating antibodies on levels of HIV provirus that persist during ART in larger, more diverse cohorts are warranted.

## Figures and Tables

**Table 1:** Cohort Descriptive Statistics.

N = 72	Median	[Min, Max]
Age at Enrollment		
Months	59	15.5, 152
Years	4.9	1.3, 12.7
Viral Load (log_10_ c/mL)	5.96	4.18, 6.96
CD4 %	6.3	0.70, 73.4
CD4 Count (cells/mm^3^)	354	15, 2009
	N	%
Gender		
Male	33	46%
Female	39	54%
ART Regimen		
NNRTI, NRTI	70	97.2%
NRTI	1	1.4%
NNRTI, NRTI, Protease Inhibitor	1	1.4%

**Abbreviations:** Min, minimum; Max, maximum; c/mL, copies/mL; NNRTI, non-nucleoside reverse transcriptase inhibitor; NRTI, nucleoside reverse transcriptase inhibitor.

## Data Availability

The data are available upon request from the corresponding author.

## Supplementary Materials

The following supporting information can be downloaded at: https://www.mdpi.com/article/10.3390/v15102055/s1, Figure S1. ADCC Measures Using Different Env Antigens are Highly Correlated. (A) Pearson correlation of log_10_ ADCC activity measured against the Clade A BG505 gp120 antigen (x-axis) and the Clade A/D BL035 gp120 antigen (y-axis); (B) Pearson correlation of log_10_ gp120-specific ADCC activity (x-axis) and log_10_ gp41-specific ADCC activity (y-axis). SFigure S2. Comparing HIV Provirus Levels in ADCC High Versus Low Groups. Unpaired *t* test of log_10_ mean HIV proviral copies between “ADCC Strong” and “ADCC Weak” groups. Participants with ADCC activity at, or above, the cohort 75th percentile were labeled “ADCC Strong” (n = 18) with those at, or below, the cohort 25th percentile labeled as “ADCC Weak” (n = 18). (A–C) Stratification based on gp120-specific ADCC activity. (A) Total HIV provirus; (B) Intact HIV provirus; (C) Defective HIV provirus; (D–F) Stratification based on gp41-specific ADCC activity. (D) Total HIV provirus; (E) Intact HIV provirus; (F) Defective HIV provirus. * denotes a *p*-value ≤ 0.05. Table S1. Linear Regression Models.
