# Supplementary material for: Higher HIV-1 Env gp120-Specific Antibody-Dependent Cellular Cytotoxicity (ADCC) Activity Is Associated with Lower Levels of Defective HIV-1 Provirus
Source: Viruses. 2023 Oct 6;15(10):2055. doi: 10.3390/v15102055 (PMC10611199; doi:10.3390/v15102055)
Supplement: Supplementary file 1 [file viruses-15-02055-s001.zip › viruses-2633101-supplementary.pdf]

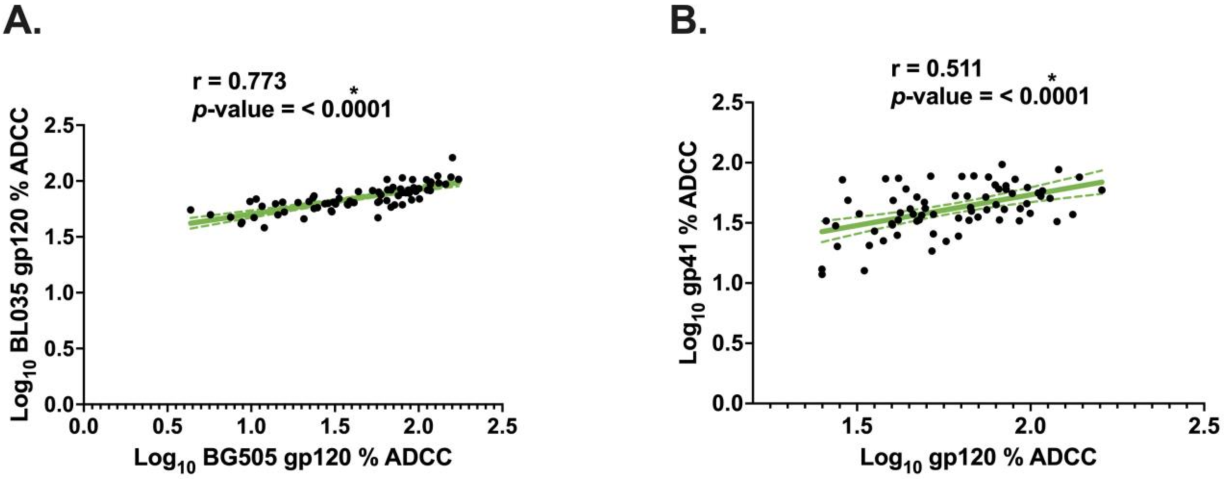

Supplementary Figure S1. Correlations of ADCC Measures. **(A)** Pearson correlation of $\log_{10}$ ADCC activity measured using the Clade A BG505 gp120 antigen and the Clade A/D BL035 gp120 antigen; **(B)** Pearson correlation of $\log_{10}$ gp120-specific ADCC activity and $\log_{10}$ gp41-specific ADCC activity. *denotes a *p*-value ≤ 0.05.

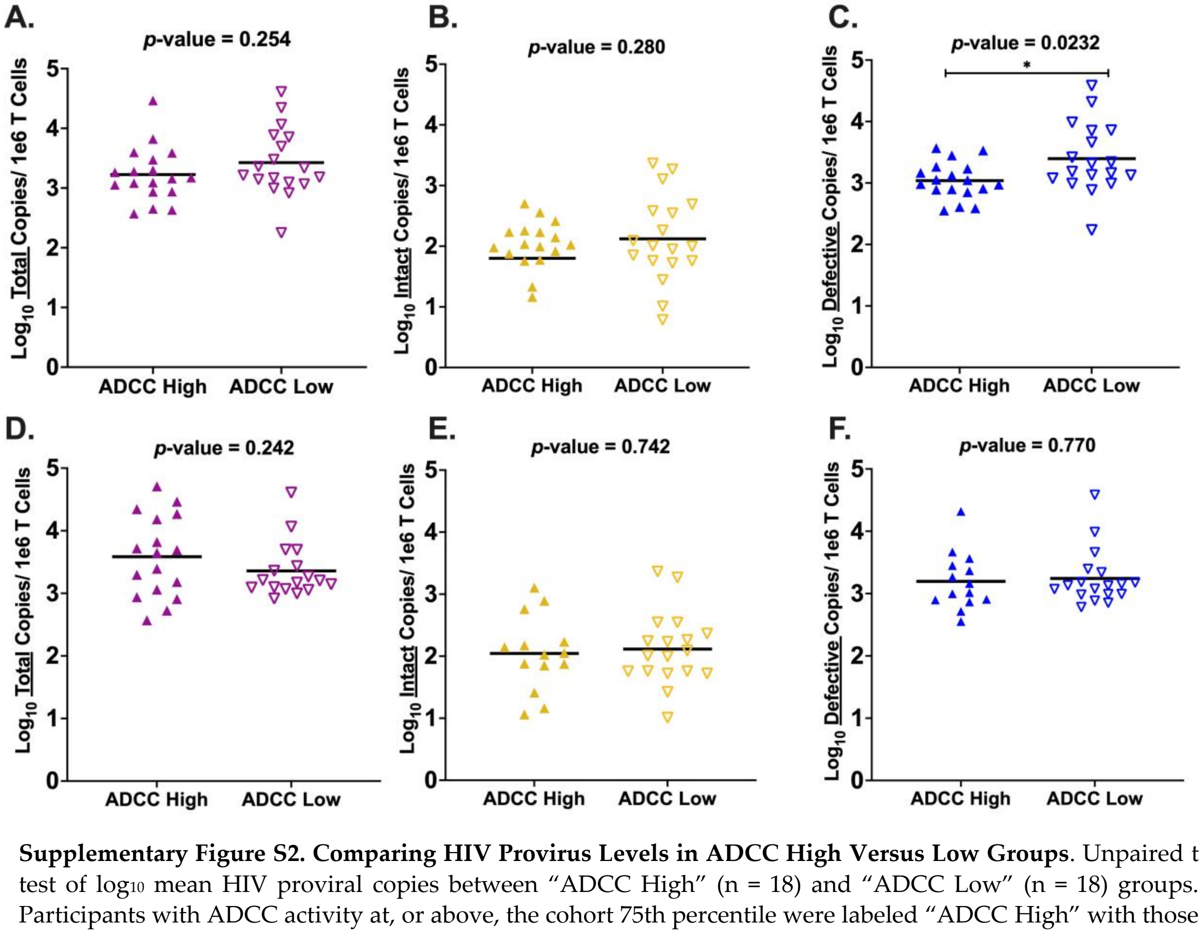

Supplementary Figure S2. Comparing HIV Provirus Levels in ADCC High Versus Low Groups. Unpaired t test of $\log_{10}$ mean HIV proviral copies between "ADCC High" (n = 18) and "ADCC Low" (n = 18) groups. Participants with ADCC activity at, or above, the cohort 75th percentile were labeled "ADCC High" with those at, or below, the cohort 25th percentile labeled as "ADCC Low." **(A-C)** Stratification based on gp120-specific ADCC activity. **(A)** Total HIV provirus; **(B)** Intact HIV provirus; **(C)** Defective HIV provirus; **(D-F)** Stratification based on gp41-specific ADCC activity. **(D)** Total HIV provirus; **(E)** Intact HIV provirus; **(F)** Defective HIV provirus. Different colors in the figures designate which proviral category is being analyzed: purple: total; yellow: intact; blue: defective. In all figures, the ADCC High group is denoted by filled in triangles pointing upward, with the ADCC Low group denoted by empty triangles pointing downward. *denotes a *p*-value ≤ 0.05.

Supplementary Table S1. Linear Regression Models.

Intact HIV Provirus	Mean (SD)	95% Confidence Interval	Univariate Coefficient (95% CI)	p-value	Full Model Multivariable Coefficient (95% CI)	p-value	Backward Selected Model Coefficient (95% CI)	p-value
Intact HIV Provirus (c/1e6 CD4 cells)	1.81 (1.03)	(1.55, 2.07)						
Age (Months) at Enrollment	64 (35)	(50.55, 77.45)	0.0040 (-0.0037, 0.0113)	0.3266				
HIV RNA (log_{10} c/mL)	5.84 (0.70)	(5.68, 6.00)	-0.0680 (-0.4555, 0.3196)	0.7323				
% CD4	10 (11)	(6.47, 13.54)	-0.0019 (-0.0259, 0.0221)	0.8761				
CD4 Count (cells/mm^3)	437 (459)	(308.81, 565.19)	-0.0004 (-0.0009, 0.0002)	0.2225				
Average % ADCC (gp120)	1.79 (0.19)	(1.77, 1.81)	-0.2012 (-1.5118, 1.1094)	0.7645	0.2354 (-1.3407, 1.8114)	0.7660	-0.0471 (-1.5270, 1.4328)*	0.9490
Average % ADCC (gp41)	1.62 (0.20)	(1.60, 1.64)	-0.2646 (-1.5404, 1.0112)	0.6857	-0.1641 (-1.6060, 1.2779)	0.8200	-0.1873 (-1.5810, 1.2064)*	0.7890

*Model includes only exposure of interest and age at enrollment (months).
**p-value <0.05

Total HIV Provirus	Mean (SD)	95% Confidence Interval	Univariate Coefficient (95% CI)	p-value	Full Model Multivariable Coefficient (95% CI)	p-value	Backward Selected Model Coefficient (95% CI)	p-value
Total HIV Provirus (c/1e6 CD4 cells)	3.35 (0.54)	(3.22, 3.48)						
Age (Months) at Enrollment	64 (35)	(50.55, 77.45)	0.0021 (-0.0016, 0.0058)	0.2677				
HIV RNA (log_{10} c/mL)	5.84 (0.70)	(5.68, 6.00)	-0.1221 (-0.3087, 0.0645)	0.2050				
% CD4	10 (11)	(6.47, 13.54)	0.0021 (-0.0100, 0.0142)	0.7391				
CD4 Count (cells/mm^3)	437 (459)	(308.81, 565.19)	-3.3e-05 (-0.0003, 0.0003)	0.8190				
Average % ADCC (gp120)	1.79 (0.19)	(1.77, 1.81)	-0.6485 (-1.3103, -0.0134)	0.0589	-0.6560 (-1.4260, 0.1139)	0.0935	-0.5137 (-1.2452, 0.2177)*	0.1650
Average % ADCC (gp41)	1.62 (0.20)	(1.60, 1.64)	0.2995 (-0.3355, 0.9345)	0.3585	0.2278 (-0.4790, 0.9354)	0.5221	0.3250 (-0.3474, 0.9975)*	0.3380

*Model includes only exposure of interest and age at enrollment (months).
**p-value <0.05

Defective HIV Provirus	Mean (SD)	95% Confidence Interval	Univariate Coefficient (95% CI)	p-value	Full Model Multivariable Coefficient (95% CI)	p-value	Backward Selected Model Coefficient (95% CI)	p-value
Defective HIV Provirus (c/1e6 CD4 cells)	3.18 (0.43)	(3.08, 3.28)						
Age (Months) at Enrollment	64 (35)	(50.55, 77.45)	0.0018 (-0.0013, 0.0049)	0.2660				
HIV RNA (log_{10} c/mL)	5.84 (0.70)	(5.68, 6.00)	-0.0340 (-0.1922, 0.1243)	0.6755				
% CD4	10 (11)	(6.47, 13.54)	0.0004 (-0.0094, 0.0103)	0.9288				
CD4 Count (cells/mm^3)	437 (459)	(308.81, 565.19)	-9.1e-05 (-0.0003, 0.0001)	0.4501				
Average % ADCC (gp120)	1.79 (0.19)	(1.77, 1.81)	-0.6349 (-1.1570, -0.1129)	0.0202**	-0.0515 (-1.1430, 0.1129)	0.106	-0.5882 (-1.1526, -0.0239)*	0.0414**
Average % ADCC (gp41)	1.62 (0.20)	(1.60, 1.64)	-0.2336 (-0.7614, 0.2942)	0.3890	-0.2245 (-0.8100, 0.3611)	0.4450	-0.2146 (-0.7784, 0.3493)*	0.4490

*Model includes only exposure of interest and age at enrollment (months).
**p-value <0.05

Supplementary Table S2. Key Resources

Reagent or Resource	Source	Identifier
Human Plasma or PBMC from PAD Cohort Participants	Wamalwa et al., JIAIDS 2009 [92]	N/A
Polyclonal Anti-Human Immunodeficiency Virus Immune Globulin, Pooled Inactivated Human Sera (HIVIG)	NIH AIDS Reagent Program	Catalog #3957
Human Negative Control Serum	NIH AIDS Reagent Program	Catalog # 2411
Human: CEM NKR	NIH AIDS Reagent Program	Catalog # 458; RRID:CVCL_X622
BG505.W6M.ENV.B1 (BG505) gp120	Cambridge Biologics, Brookline, MA, USA	Cat#01-01-1028
BL035.W6M.ENV.C1 (BG035) gp120	Immune Technology Corporation, New York, NY, USA	Cat#IT-001-115p
ZA.1197MB gp41	Immune Technology Corporation, New York, NY, USA	Cat# IT-001-0052p
PKH26 Red Fluorescent Cell Linker Kit	Sigma Aldrich, Saint Louis, MO, USA	Cat#MINI26-1KT
CellVue Claret Far Red Cell Linker dye	Sigma Aldrich, Saint Louis, MO, USA	Cat #MINCLARET-1KT
Vybrant CFDA SE. Cell Tracer Kit	Invitrogen, Waltham, MA, USA	Cat#V12883
FlowJo v9.9	TreeStar	RRID: SCR_008520
Excel v16.76	Microsoft	RRID: SCR_016137
Prism 9.5c	GraphPad	RRID: SCR_002798
